# Assessing the Risk of Vaccine-derived Outbreaks After Reintroduction of Oral Poliovirus Vaccine in Postcessation Settings

**DOI:** 10.1093/cid/ciy605

**Published:** 2018-10-30

**Authors:** Rui Fu, Jonathan Altamirano, Clea C Sarnquist, Yvonne A Maldonado, Jason R Andrews

**Affiliations:** 1 Department of Management Science and Engineering, Stanford University, California; 2 Division of Pediatric Infectious Diseases, California; 3 Division of Infectious Diseases and Geographic Medicine, Stanford University School of Medicine, California

**Keywords:** infectious diseases, oral polio vaccine, global OPV cessation, circulating vaccine-derived poliovirus

## Abstract

**Background:**

The Polio Eradication and Endgame Strategic Plan 2013–2018 calls for the gradual withdrawal of oral poliovirus vaccine (OPV) from routine immunization. We aimed to quantify the transmission potential of Sabin strains from OPV when it is reintroduced, accidentally or deliberately, in a community vaccinated with inactivated poliovirus vaccine alone.

**Methods:**

We built an individual-based stochastic epidemiological model that allows independent spread of 3 Sabin serotypes and differential transmission rates within versus between households. Model parameters were estimated by fitting to data from a prospective cohort in Mexico. We calculated the effective reproductive number for the Mexico cohort and simulated scenarios of Sabin strain resurgence under postcessation conditions, projecting the risk of prolonged circulation, which could lead to circulating vaccine-derived poliovirus (cVDPV).

**Results:**

The estimated effective reproductive number for naturally infected individuals was about 1 for Sabin 2 and Sabin 3 (OPV2 and OPV3) in a postcessation setting. Most transmission events occurred between households. We estimated the probability of circulation for >9 months to be (1) <<1% for all 3 serotypes when 90% of children <5 years of age were vaccinated in a hypothetical outbreak control campaign; (2) 45% and 24% for Sabin 2 and Sabin 3, respectively, when vaccine coverage dropped to 10%; (3) 37% and 8% for Sabin 2 and Sabin 3, respectively, when a single active shedder appeared in a community.

**Conclusions:**

Critical factors determining the risk of cVDPV emergence are the scale at which OPV is reintroduced and the between-household transmission rate for poliovirus, with intermediate values posing the greatest risk.

As polio eradication efforts continue globally, the goal of polio eradication may soon be achieved. Over the past 3 years, there has been a decrease from 87 cases of wild-type poliovirus (WPV) in 2015 to only 22 cases in 2017 [1]. Currently, WPV serotype 1 is the only serotype believed to still be in circulation, and only in Pakistan and Afghanistan [2]. By contrast, WPV serotype 2 was declared eradicated in 2015, the first pathogen globally eradicated since smallpox in 1980, and WPV serotype 3 has not been detected since November 2012.

The success of the Global Polio Eradication Initiative can be attributed to the widespread use of the Sabin oral poliovirus vaccine (OPV). OPV is particularly effective in low- and middle-income settings, owing to its ease of administration, low cost, and ability to confer community immunization through fecal-oral transmission to unvaccinated household and community contacts [3]. However, continued use of OPV is now complicating eradication efforts, because prolonged circulation of OPV can lead to the development of OPV variants known to cause polio. Vaccine associated paralytic polio is estimated to cause 2–4 cases per 1000000 live births per year in countries vaccinating with OPV [4]. In addition, extended replication and mutation of OPV can lead to genetically divergent vaccine-derived polioviruses (VDPVs), defined as 1% divergence from the parent strain for OPV serotypes 1 and 3 (OPV1 and OPV3) and 0.6% divergence from the parent strain of OPV serotype 2 (OPV2) [5, 6]. Circulating VDPVs (cVDPVs), viruses with evidence of community circulation, can cause paralysis that is phenotypically indistinguishable from WPV [5]. In 2017, a total of 91 cases of cVDPVs were identified, particularly after outbreaks in Syria and the Democratic Republic of Congo [1]. In response to the growing concern of cVDPVs, the Global Polio Eradication Initiative has called for the replacement of OPV with inactivated poliovirus vaccine (IPV), beginning with the withdrawal of OPV2 in April 2016, and the introduction of ≥1 dose of IPV into global vaccination schedules by 2016 [7, 8].

With this transition in mind, a variety of different models have looked to explore Sabin virus circulation after stopping routine use of OPV and the probability of emergence of cVDPVs in the postcessation and posteradication world [9–13]. Of particular interest in a number of these models is the circulation of Sabin 2 [9, 10], owing to the concern for reemergence of cVDPV serotype 2, because >90% of all cVDPV cases to date have been caused by Sabin 2 [2]. We developed an individual-based stochastic epidemiological model to look at the effective reproductive number of each Sabin serotype in a Mexican cohort located in 3 semiurban, geographically isolated indigenous villages. We aimed to estimate within- and between-household transmission rates, simulate this population’s response to reintroduction of OPV in the postcessation future, and quantify the magnitude and duration of potential community circulation of Sabin virus.

## METHODS

### Mexico Data

**Figure 1.**
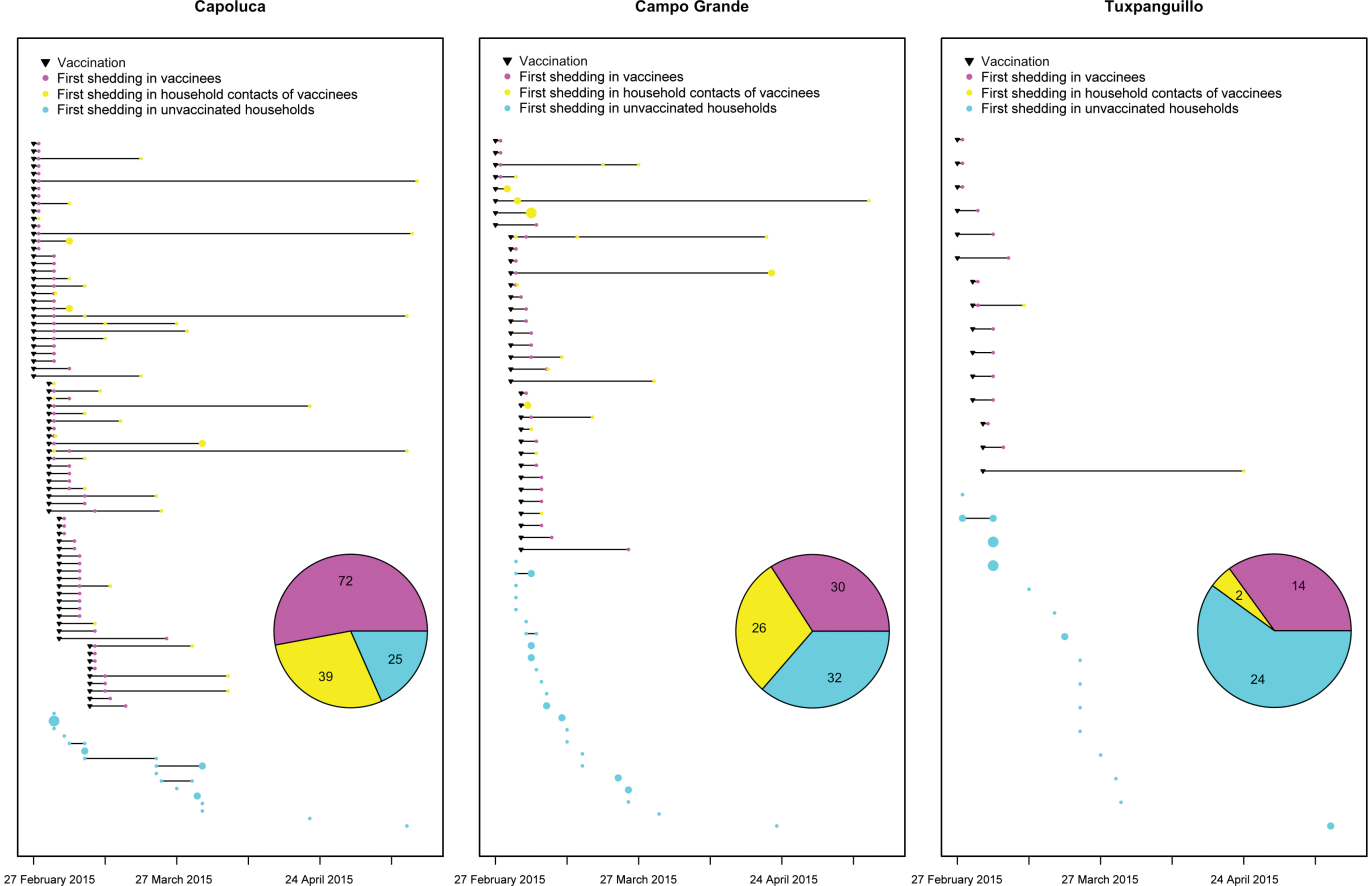
Vaccinations and first shedding events by household. A shedding event was defined as the presence of ≥1 serotype in a stool sample, and only the first shedding event for each individual is displayed. Each row corresponds to a household, and the size of points is scaled to case numbers. Pie chart shows the cumulative number of shedding events in vaccinees, household contacts of vaccinees, and unvaccinated households. (Households that were enrolled in the Mexico study but provided no positive stool samples throughout the study period are not shown.)

In 2015, a prospective observational study was conducted in 3 indigenous localities (Capoluca, Campo Grande, and Tuxpanguillo) in Orizaba, Veracruz, Mexico [14]. In each community, approximately 150 households were enrolled, and each community cluster received a different amount of OPV vaccination as part of the study: 70% of children aged <5 years in enrolled households in Capoluca, 30% in Campo Grande, and 10% in Tuxpanguillo. When enrollment began in February 2015, a total of 155 households were randomized to receive OPV, of 466 households included across the 3 localities. No other households in these 3 communities received OPV while the study was conducted. Only 1 child from each of the 155 households received OPV. In total, 10 stool samples were scheduled for collection from each member of enrolled households (irrespective of vaccination assignment), including 1 baseline sample collected before the vaccination campaign, and samples collected 1, 4, 7, 10, 14, 21, 28, 51, and 71 days after vaccination. All samples were analyzed by means of real-time polymerase chain reaction to detect the presence of 3 OPV strains, following methods described elsewhere [15]. We illustrate the time series of OPV vaccinations as well as first shedding events by household in Figure 1.

### Mathematical Model

We built a discrete time stochastic model in which 3 Sabin serotypes spread within a community independently. Per serotype, individuals progress through a series of 4 states over time: susceptible, latently infected, infectious, and recovered. We assumed that fecal shedding occurs only at the infectious stage. Vaccination and natural infection can both result in a transition from susceptible state to latent state. Individuals then progress from latent state to infectious state at a rate given as the reciprocal of the average latency duration. Similarly, individuals recover from infection at a rate that is the reciprocal of the average shedding duration. We allowed latency and shedding durations to depend on serotype, prechallenge immunization history (IPV only or OPV exposed), and source of poliovirus (vaccine or natural infection). Several studies reported shedding durations of children challenged with OPV [16], though we estimated these durations directly from the data. This is due to the concern that these studies were conducted in different regions and eras, so the health condition of their participants may differ considerably from the children in the Mexico study.

Person-to-person fecal-oral exposure was assumed to be the sole route of transmission in our model. Differences in daily fecal exposure to household contacts versus community members are incorporated by differential transmission rates within versus between households. The hazard of infection for a susceptible individual is given by the sum of within- and between-household probabilities of transmission. The within-household hazard is calculated by multiplying the within-household transmission rate by the number of persons shedding within this individual’s household, and the between-household hazard is calculated in a similar way. We allowed transmission rates to vary by community, reflecting differential effective contact rates and sanitation conditions, and by serotype, reflecting serotype-specific transmissibility. To limit the number of unknown model parameters, we assumed that relative differences in transmission between strains are consistent across communities.

### Model Fitting and Postcessation Simulations

We estimated model parameters, including latency and shedding durations, and transmission rates, with a likelihood-free inference technique called *approximate Bayesian computation* [17]. Further details are provided in the Supplementary Material. We then used the estimates of parameters for simulating 2 probable scenarios wherein OPV is reintroduced 5 years after cessation. First, we examined community circulation of Sabin virus when OPV is deployed in a hypothetical outbreak response. All children <5 years old are eligible for vaccination and are selected uniformly at random to receive the vaccine. We varied vaccination coverage rate within the range of 10%–90% and performed 10 000 stochastic simulations at each coverage level. All vaccination occurred on simulated day 0, and a simulation was ended when every individual in the population was either susceptible to or recovered from all 3 Sabin serotypes.

The second scenario involves the appearance of a single active case that sheds Sabin virus in a community, potentially setting off silent circulation. The active case could be an importation from an OPV-using region or an inadvertent leak from laboratory that maintains stocks of OPV. Ten thousand simulations were performed, and the cumulative incidence of infections and the duration of persistent transmission for all those simulations were presented. We defined cVDPV risk as the proportion of simulations in which Sabin virus persisted for >9 months after introduction [18].

In simulations, children <5 year of age were born after cessation and vaccinated only with IPV. In alignment with estimates from the literature [16], we assumed vaccine recipients to shed 20 days on average after OPV challenge, whereas naturally infected individuals shed for half as long. We explored sensitivity of model predictions to this assumption. As to individuals aged >5 years who were born before cessation, we expected their intestinal immunity (induced by WPV and/or OPV) to have gradually waned; we thus initialized each of them to the susceptible state with a probability of 0.8, and to the recovered state with the remaining probability. To address the uncertainty in speed of waning, we also varied this probability in the sensitivity analysis.

## RESULTS

### Serotype Differences

**Figure 2.**
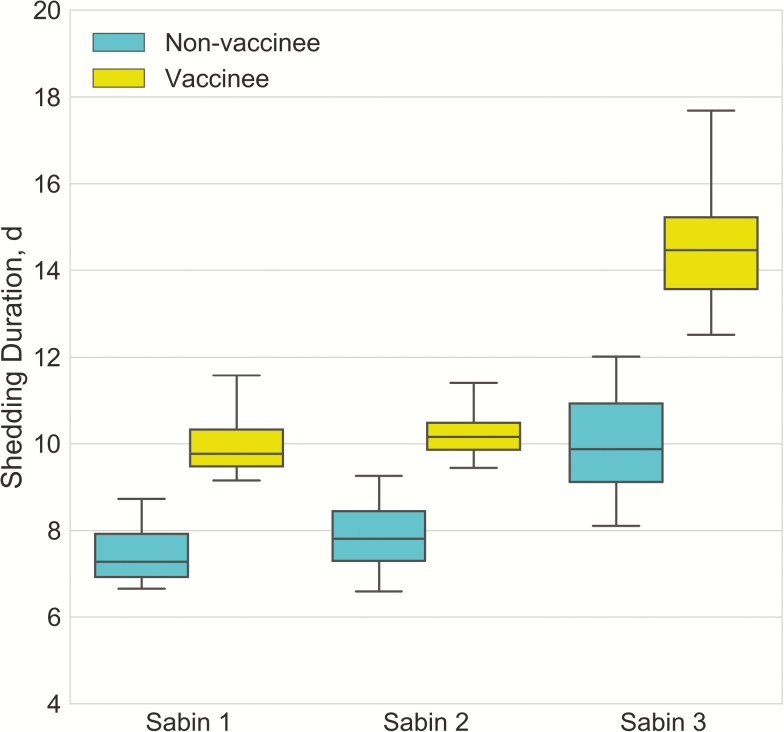
Shedding duration in Mexican study cohort by Sabin serotype, in vaccine recipients and naturally infected individuals.

With Sabin 2 used as the reference, the estimated relative infectivities of Sabin 1 and Sabin 3 were 0.36 (95% credible interval [CI], .21–.63) and 0.75 (.58–.90), respectively. This agrees with the finding in previous studies that Sabin 2 is the most transmissible among 3 serotypes [19–21]. Figure 2 shows the estimated shedding duration in the Mexican cohort, by serotype and source of infection. Vaccinees tended to shed longer than naturally infected individuals, possibly owing to the higher dose acquired from vaccine compared with from fecal-oral exposure. As to serotype, Sabin 3 led to longer shedding than Sabin 1 and 2 for both vaccinees and nonvaccinees.

### Effective Reproductive Number

**Figure 3.**
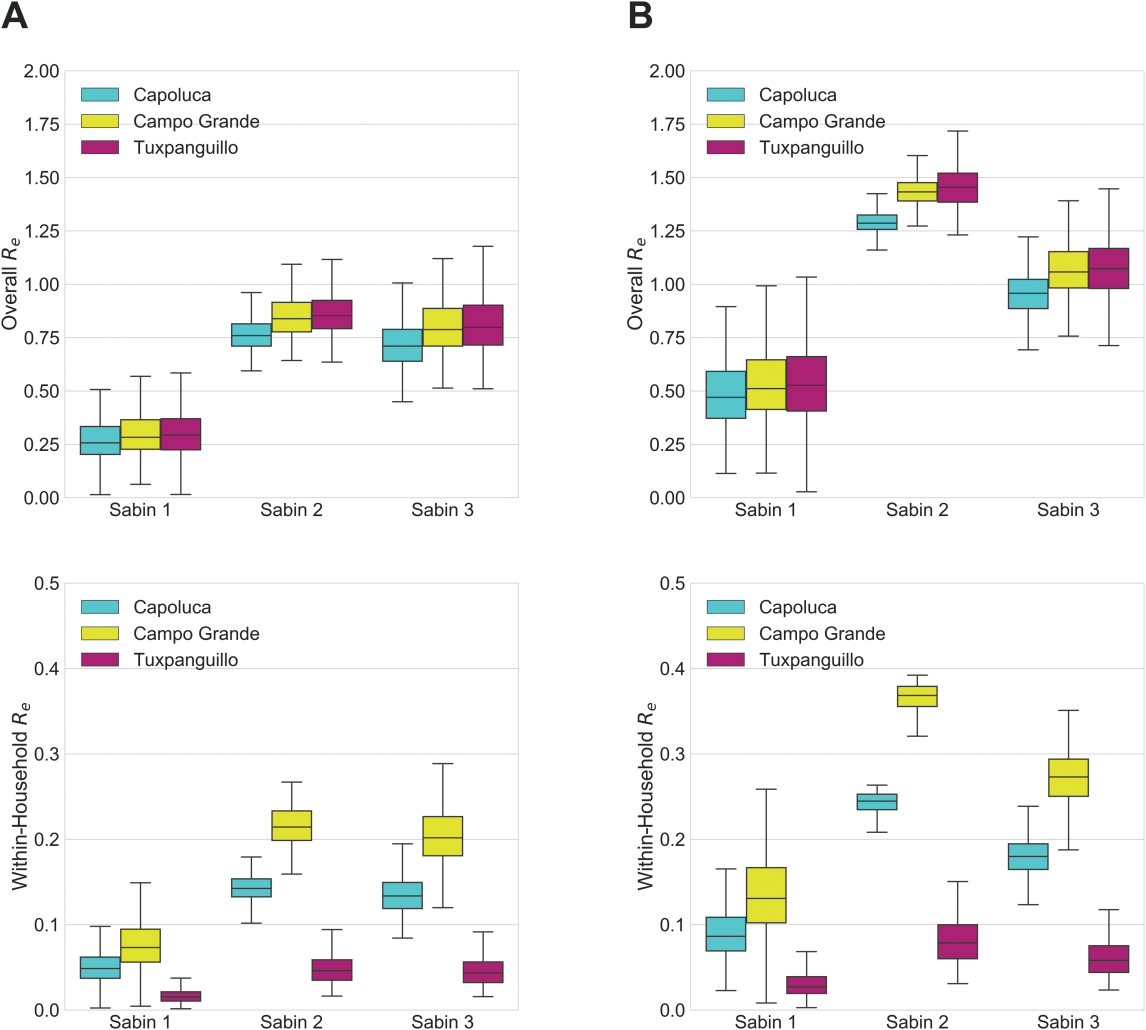
Effective reproductive number for nonvaccinees. Overall and within-household effective reproductive numbers (*R*_*e*_) values estimated at the onset of the Mexico study (*A*) and 5 years after cessation of oral poliovirus vaccine (*B*).

The effective reproductive number, *R*_*e*_, was calculated from multiplying the average duration of infectiousness with transmission rate and number of susceptible individuals. Figure 3 shows the overall and within-household *R*_*e*_ estimated for nonvaccinees in the Mexico study and in the same cohort in postcessation settings. At the onset of the Mexico study, the expected number of secondary cases caused by a vaccinee was 0.35 (95% CI, .16–.60), 0.98 (.88–1.12), and 1.03 (.80–1.36) for Sabin 1, 2, and 3 in Capoluca, the community with the greatest density of data. For nonvaccinees, the effective reproductive numbers for Sabin 1, 2 and 3 were 0.26 (95% CI, .12–.46), 0.76 (.64–.90), and 0.71 (.53–.96), respectively. Overall *R*_*e*_ values are similar across communities. It is apparent that the majority of secondary cases caused by an infectious individual were not in his or her household; this was particularly evident in Tuxpanguillo, where 24 of the 26 nonvaccinees who were ever observed to excrete Sabin strains during the study were from unvaccinated households.

Although per-person transmission rates are higher within households, the majority of transmissions for all serotypes and communities occurred between households, owing to the larger number of potential exposures. It is important to note that the sampling strategy was such that household members of vaccinees were overrepresented, because all vaccine households and only a small fraction of nonvaccine households were followed up. Therefore, the observed secondary cases in nonvaccine households were a fraction of all such infections. This sampling fraction was accounted for in the model and explains the high between-household transmission estimate. In postcessation Capoluca, the expected numbers of secondary cases generated by a nonvaccinee were 0.47 (95% CI, .22–.82), 1.29 (1.20–1.40), and 0.96 (.75–1.21) for the 3 serotypes. The increase in estimated *R*_*e*_ was due to the larger susceptible pool as well as the longer shedding duration in children who have never been exposed to OPV.

### Postcessation Scenario: Outbreak Response

**Figure 4.**
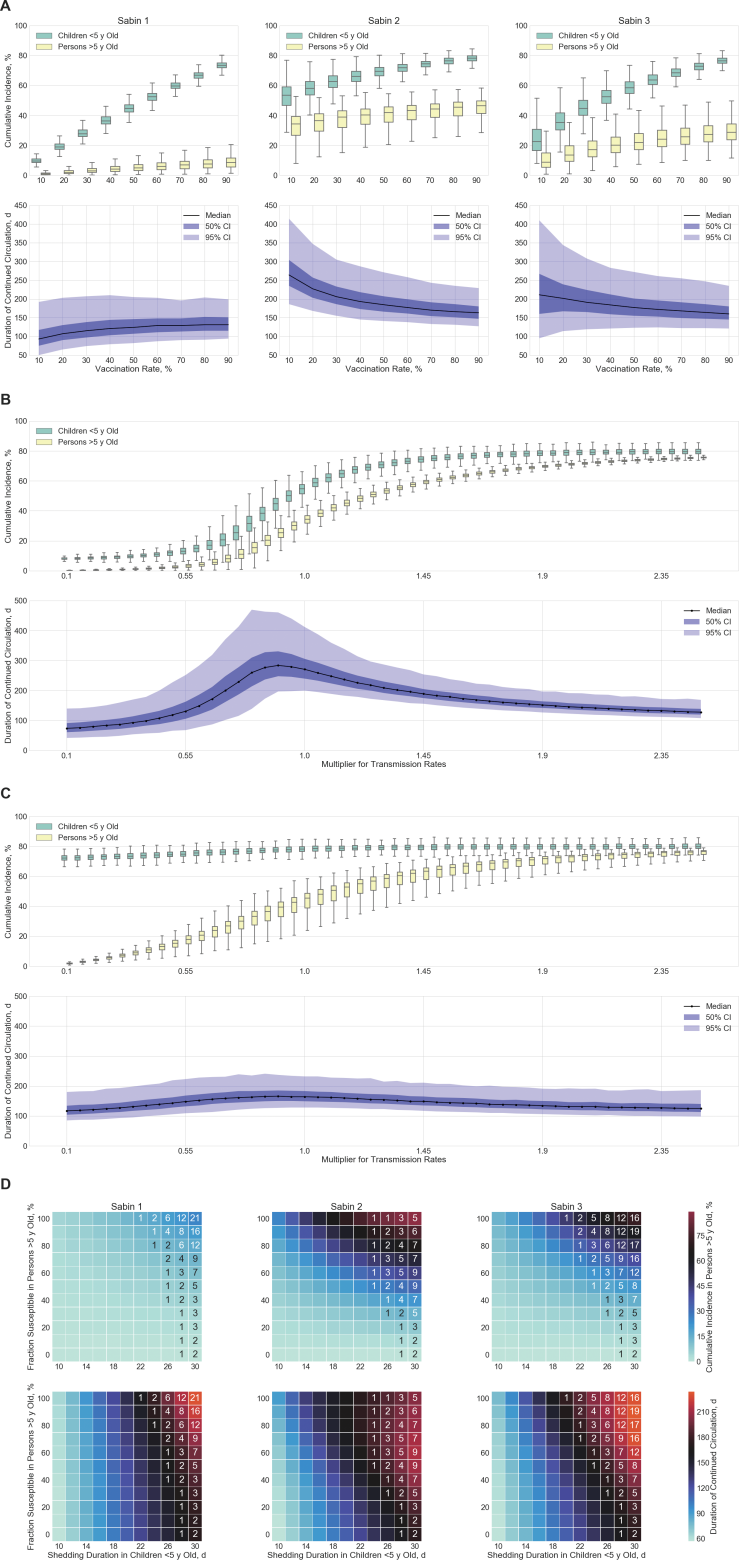
Simulated outbreak response using oral poliovirus vaccine (OPV) in Capoluca 5 years after cessation. *A,* Predicted cumulative incidence and duration of continued transmission by Sabin serotype, according to vaccination rate among children <5 years of age. *B,* Sensitivity analysis on transmission rates of Sabin 2 with vaccine coverage fixed at 10%; the same multiplier is applied to within-household and between-household transmission rates. *C,* Sensitivity analysis on transmission rates of Sabin 2 with vaccine coverage fixed at 90%. *D,* Sensitivity analysis on shedding duration in children <5 years of age (born after cessation and vaccinated with inactivated poliovirus vaccine alone in routine immunization) and fraction susceptible to infection in persons >5 years of age (born before cessation and exposed to OPV viruses before). Grid color indicates median of the simulation outputs, and annotations represent nonzero probabilities (percentages) of OPV circulating for >9 months in a community. Abbreviation: CI, credible interval.

Figure 4A presents the cumulative incidence and duration of circulation when a hypothetical outbreak response using OPV is launched in Capoluca (results for Campo Grande and Tuxpanguillo are shown in Supplementary Figures 1 and 2). The cumulative incidence in both age groups increased linearly with vaccine coverage, though the slopes for the 3 serotypes were different, reflecting their relative transmissibility. This disparity in transmissibility is manifested in different patterns of Sabin virus persistence. If the vaccination rate was increased from 10% to 90%, the median of circulation duration grew from 93 to 131 days for Sabin 1 and dropped from 264 to 163 days for Sabin 2 and from 211 to 160 days for Sabin 3. Specifically, cVDPV risk (defined as the proportion of simulations in which transmission persisted >9 months) for Sabin 1 was <<1% at any vaccination rate, whereas the risk for Sabin 2 and 3 peaks at 45% and 24%, respectively, when 10% of children <5 years of age are vaccinated.

We conducted univariate sensitivity analysis to assess the impact of transmission rates on persistence of Sabin 2, varying these rate between 10% and 250% of the estimated rates in the study communities. This multiplier could reflect site-specific differences in the effective contact rate, as well as differences in the infectivity of strains. For example, improvement of sanitation conditions nudges the multiplier to <1, whereas Sabin 2 that has reverted to full virulence may be associated with a large multiplier owing to its greater transmissibility. At a fixed vaccination rate of 10%, the duration of Sabin 2 circulation in the community peaked at a transmission rate approximately 90% of that observed in the study communities (Figure 4B), a scenario in which 60% of simulations involved circulation >9 months.

As expected, high transmission rates lead to rapid spread of OPV viruses to unvaccinated individuals, which in turn depletes the susceptible population and undermines the potential for sustained circulation of Sabin strains. On the other hand, a sufficiently small force of infection can also prevent long-term viral transmission through extinction of infection. Figure 4C shows the output of simulations when OPV is distributed to 90% of eligible children during an outbreak response. Cumulative incidence among young children was minimally affected by transmission rate, because most of these children acquired Sabin viruses through vaccination, whereas the incidence in the older group was significantly influenced by the transmission rate. The duration of persistence exhibited low variance and was <250 days in all simulated runs.

We then conducted a bivariable sensitivity analysis to address the wide uncertainty about the fraction susceptible to OPV viruses in persons born before cessation and the shedding duration in IPV-only cohort. Fixing the vaccination rate at 90% to provide sufficient protection for young children, we varied the values of these 2 parameters. The cumulative incidence in persons >5 years of age and the circulation duration both increased as susceptibility to infection and shedding duration increased. Of note, serotype-specific maximum risks of cVDPV were 21%, 9%, and 19% respectively, all achieved when the shedding duration in the IPV-only cohort had the largest value allowed (that is, 30 days). Compared with Sabin 2, the relatively low transmissibility of Sabin 1 and 3 moderated the speed of spread and prevented early exhaustion of susceptible individuals, allowing these strains to persist even longer in the community. Our result demonstrates that the waning immunity in persons born before cessation gives rise to an appreciable pool of susceptibles, which could potentially nurture prolonged circulation of OPV viruses if the average shedding duration in vaccinated children is >20 days.

### Postcessation Scenario: Silent Circulation Triggered By 1 Shedding Case

**Figure 5.**
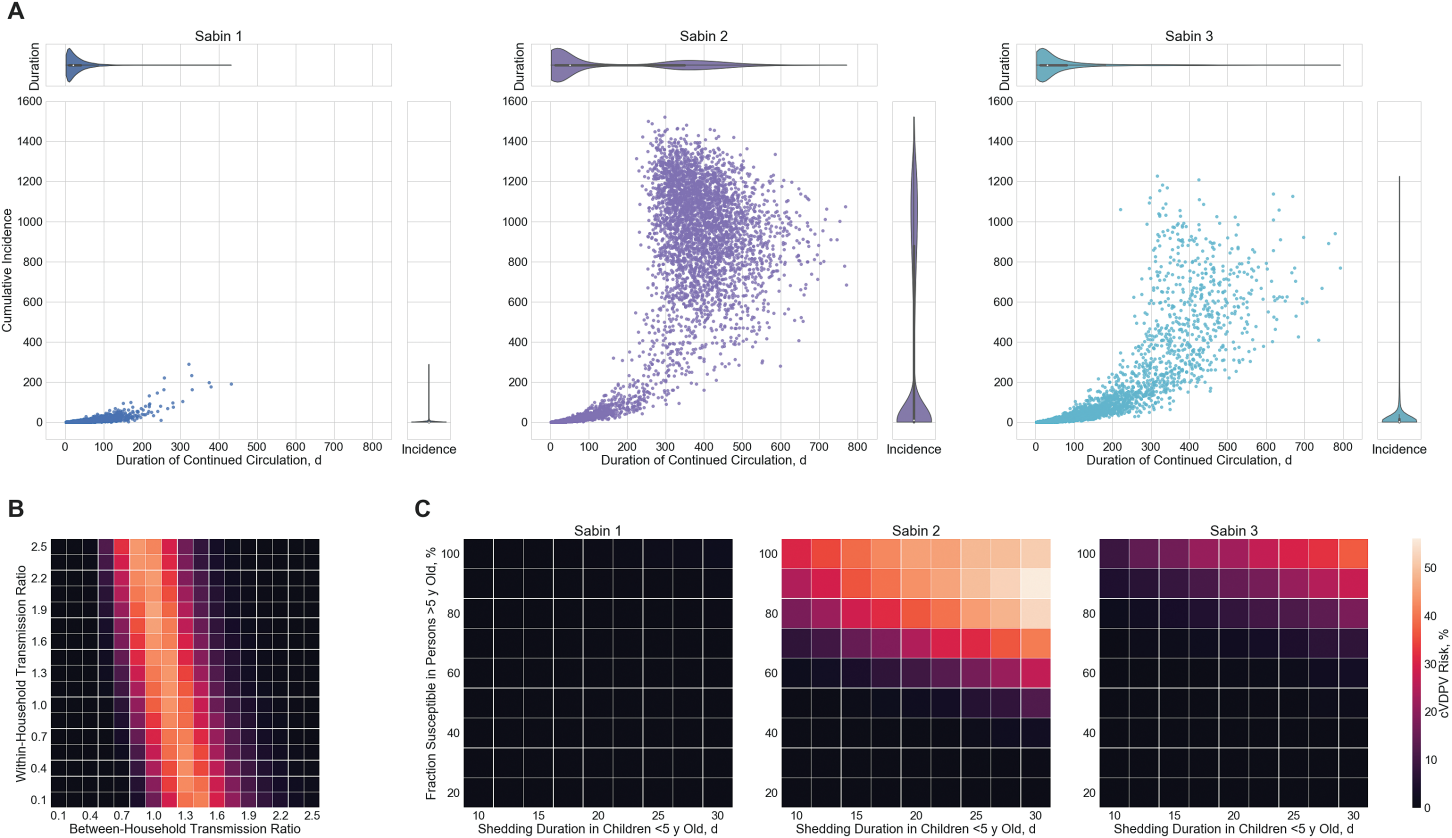
Simulated silent circulation triggered by 1 infectious case shedding Sabin virus in Capoluca 5 years after cessation. *A,* Predicted magnitude and duration of continued transmission by serotype. Scatterplots show results of each simulation; violin plots, marginal distributions of duration and total cases. *B,* Sensitivity analysis on within- and between-household transmission rates of Sabin 2. Grid color indicates probability of Sabin virus circulating for >9 months in a community. *C,* Sensitivity analysis on shedding duration in children <5 years of age and fraction susceptible to infection in persons >5 years of age. Abbreviation: cVDPV, circulating vaccine-derived poliovirus.

Figure 5A illustrates the magnitude and duration of simulated OPV virus circulation in Capoluca after the appearance of a case actively excreting Sabin strain. Results for Campo Grande and Tuxpanguillo are similar and are shown in Supplementary Figures 3 and 4. The median durations of circulation were 21, 51, and 30 days for the 3 Sabin serotypes, and the median cumulative numbers of secondary infections were 1, 9, and 3. This seems to suggest that Sabin virus is likely to self-extinguish when only 1 active case is released into the community; however, the expected duration of transmission was bimodal, and there was a nonnegligible risk of persistence >9 months for Sabin 2 (36.5%) and Sabin 3 (7.8%).

We evaluated how change in transmission rates would affect these results. For these simulations, we allowed between-household and within-household transmission rates of Sabin 2 to differ. Figure 5B shows that cVDPV2 risk is mainly determined by the multiplier relating to between-household transmission rate. Low rates of between-household transmission lead to extinction of virus transmission without prolonged circulation, whereas high transmission rates lead to rapid infection of the entire susceptible population; intermediate values pose the highest risk of prolonged circulation. In addition, we examined the sensitivity of results to the assumed value of shedding duration in children <5 years old with IPV-only immunization history and the susceptibility fraction in persons aged >5 years. Figure 5C shows that cVDPV risk generally increased with these 2 variables, and the serotype-specific maximums were 1.6%, 56.1%, and 37.0%. It is worth mentioning that cVDPV risk dropped to <1% for all serotypes when ≤50% of the population born before cessation was susceptible, and vaccine recipients born after cessation excreted OPV virus for an average of ≤15 days.

## DISCUSSION

Removal of OPV from routine immunization is in progress globally to achieve the goal of eventual elimination of poliovirus. However, it foreshadows a growing population of individuals without intestinal immunity, who would be susceptible to infection and shedding if OPV were to be reintroduced, either deliberately for WPV control or inadvertently through migration or laboratory-derived outbreaks. Understanding how OPV viruses may circulate in postcessation settings and the potential for cVDPV emergence is therefore critical to informing public health responses.

Using data on stool shedding and transmission from a prospective study of OPV administration in 3 Mexican communities [14], we calibrated an individual-based stochastic epidemiological model and estimated within- and between-household transmission rates for each serotype. We estimated that each nonvaccinee caused an average of 0.26, 0.76, and 0.71 secondary cases of type 1, 2, and 3 Sabin virus in Capoluca, and the estimates for the other 2 communities were similar. Because the *R*_*e*_ was <1 for nonvaccinees, transmission extinguished after a few months. We then modeled the same cohort 5 years after cessation of OPV, whereby susceptibility to intestinal infection was greater, and the *R*_*e*_ for nonvaccinees was estimated to be 0.47, 1.29 and 0.96, with prolonged circulation (>9 months) occurring for Sabin 2 and Sabin 3 in many of the simulations. These findings indicate that there may be substantial risk for cVDPV emergence if OPV were to be reintroduced in postcessation settings.

Importantly, our findings suggest if OPV is reintroduced for outbreak containment, settings with low OPV coverage may be at highest risk for prolonged circulation. We simulated a postcessation outbreak response in which children <5 years old were vaccinated with OPV. At 10% coverage, the probabilities of Sabin 2 and Sabin 3 circulating >9 months were 45% and 24%, whereas at 90% coverage the median duration of circulation was <165 days for all serotypes. Our results suggest that an aggressive response is essential to interrupt WPV or cVDPV transmission without seeding a new cVDPV outbreak. In addition, expanding the vaccination campaign to include older age groups may be necessary for preventing cVDPV outbreaks if duration of fecal excretion in children is sufficiently long.

We found the probability of prolonged outbreak, in which cVDPVs may arise, is highly sensitive to the transmission rate of OPV viruses between households. For low transmission rates, the *R*_*e*_ value is <1, and outbreaks are constrained. For high transmission rates, OPV viruses quickly spread through the community and the duration of circulation is limited by exhaustion of susceptible individuals. At intermediate transmission values, the risk of prolonged circulation is greatest. These findings may be important in risk assessment surrounding OPV use and for focusing surveillance for cVDPV emergence. Our findings concerning the overall probability of cVDPV emergence are consistent with those of a 2017 study examining the possibility of outbreak responses seeding cVDPV2 after cessation of OPV2 [18]. That study estimated this probability to exceed 50% as soon as 18 months after cessation, an estimate supported under the highest risk conditions by our model.

Another scenario we considered was a limited release of OPV in a postcessation setting. We simulated the extreme case that starts with only 1 infectious individual in each community. The probabilities of Sabin 2 and Sabin 3 persisting >9 months in Capoluca were 36.5% and 7.8%, respectively. Under higher-risk conditions, the probability reached 50% for serotype 2. These findings underscore the importance of secure containment of sources of OPV, a major current emphasis of the World Health Organization [8], as well as intensive surveillance in a postcessation era.

The interpretation of these results should be understood within the context of the limitations of the available data and assumptions of our models. We estimated transmission rates by calibrating our model to time series data on stool shedding after introduction of OPV into 3 communities, which provided a unique opportunity to estimate within- and between-household transmission. However, shedding was measured at only 10 time points and for a fraction of each community; though our model assumed that most shedding was not measured by the study, it posed challenges for parameter estimation.

Given these limitations in data, we made a number of simplifying assumptions about poliovirus natural history and transmission, consistent with previously published models [10, 13, 22], to create a relatively parsimonious model that would improve parameter identifiability and avoid overfitting [23]. We modeled only 2 age groups (<5 and >5 years), did not model assortative mixing behaviors outside the household, and assumed that intestinal immunity generated during the study period prevented reinfection during the simulation period. We assumed that transmission of the 3 Sabin strains was independent, despite the intertypic interference effects observed by many studies [24–26]. However, a model that conveys the interaction among serotypes would be more prone to overfitting due to its complexity.

In addition, we assumed that the communities were isolated without births or migration during the time horizon of our simulations. This assumption is mostly reasonable in model fitting, considering that the Mexico study spanned only 80 days, and the 3 communities were specifically chosen because they were geographically isolated from each other by a series of valleys, preventing intermixing between the villages. However, in other settings, it is likely that intercommunity transmissions may help sustain long-term viral circulation, by supplying the epidemics with susceptible individuals from contiguous communities. In such settings, our projections probably underestimate the probability of cVDPV outbreaks after cessation. Finally, generalization of our specific results to settings beyond the 3 study communities should be done with caution, because the determinants of transmission rates, such as sanitation conditions and social mixing pattern, vary considerably across geographic contexts. Nevertheless, we simulated a wide range of transmission rates in sensitivity analysis, which provides useful lower and upper bounds for the duration and magnitude of Sabin virus circulation in postcessation communities. The findings concerning vaccination coverage and the high cVDPV risk occurring in medium transmission settings may represent valuable general insights.

As the global movement toward withdrawal of OPV and replacement with IPV approaches completion, it is important to understand the risks associated with OPV reintroduction by intention or accident in communities without intestinal immunity to polioviruses. In particular, we find that the critical factors in determining the risk of cVDPV emergence are the scale at which OPV is reintroduced and the between-household transmission rate for poliovirus, with intermediate values posing the greatest risk. It is critical to consider these factors when planning outbreak responses and surveillance in the postcessation era.

## Supplementary Data

Supplementary materials are available at *Clinical Infectious Diseases* online. Consisting of data provided by the authors to benefit the reader, the posted materials are not copyedited and are the sole responsibility of the authors, so questions or comments should be addressed to the corresponding author.

## Supplementary Material

Supplemental_data

## Notes

***Financial support.*** This work was supported by the Bill & Melinda Gates Foundation (grant OPP1116910).

***Supplement sponsorship.*** This article appears as part of the supplement “Polio endgame and beyond: Vaccine choices, transmission dynamics, and surveillance implications” sponsored by The Bill & Melinda Gates Foundation.

***Potential conflicts of interest.*** All authors: No reported conflicts. All authors have submitted the ICMJE Form for Disclosure of Potential Conflicts of Interest. Conflicts that the editors consider relevant to the content of the manuscript have been disclosed.
