# Supplementary material for: Assessing the Risk of Vaccine-derived Outbreaks After Reintroduction of Oral Poliovirus Vaccine in Postcessation Settings
Source: Clin Infect Dis. 2018 Oct 30;67(Suppl 1):S26–34. doi: 10.1093/cid/ciy605 (PMC6206116; doi:10.1093/cid/ciy605)
Supplement: Supplemental_data [file ciy605_suppl_supplemental_data.docx]

**Supplementary Materials**

**Supplemental Methods**

*Model Overview*

We built a discrete time stochastic model in which three OPV serotypes spread within a community. Per serotype, individuals progress through a series of four states over time: susceptible, latently infected, infectious, and recovered. Fecal shedding only occurs at the infectious stage, and fecal-oral exposure is the sole route of transmission. Differences in daily fecal exposure to household contacts versus community members is incorporated by differential transmission rates within versus between households.

We obtained census and shedding data from a study conducted in three semi-urban communities in Mexico, 2015 [1]. The census data allows us to construct household-structured populations in our model, while the shedding data provides three instances of community circulation of OPV to which the model was fit. We estimated model parameters using a likelihood-free inference technique called Approximate Bayesian Computation (ABC), because the likelihood for a partially-observed individual-based epidemiological model is mathematically intractable. Approximate posterior distribution of the parameters is then obtained and used for simulating two scenarios when OPV is reintroduced to the same communities 5 years post-cessation.

Several simplifying assumptions were made to constrain the complexity of our disease model. First, we assumed OPV1, OPV2, and OPV3 circulate independently in a community. That is, harboring one serotype neither facilitates nor obstructs the acquisition of the other two serotypes in the same host, and infection of one serotype confers no heterotypic immunity. Second, we assumed individuals in the recovered state for some Sabin strain will not become susceptible to subsequent infection with the same strain during the simulation period. This assumption is justifiable considering (1) intestinal immunity conferred by OPV wanes over a timescale of years [2]; and (2) our model is run for 80 simulated days (equal to length of the Mexico study) in model fitting, and no longer than 800 days in post-cessation simulations. Third, as the contribution of environmental reservoir to circulation of OPV is limited [3], we did not explicitly model environmental force of infection and modeled person-to-person transmission exclusively. Lastly, our model assumed closed and isolated populations. In other words, we did not include births, deaths, or migration during the relatively short time horizon of the model.

*Natural history assumptions for OPV infection*

For any serotype of OPV, we modeled individuals as either susceptible, latently infected, infectious, or fully recovered at any point in time. In model fitting, the entire population were assumed to be susceptible at the beginning of the study. This is unlikely considering Mexico gave OPV to young children during biannual national immunization weeks, and OPV induces intestinal immunity against reinfection, nonetheless there is no seroprevalence data to inform our model of the immunologic status of each individual. In view of this difficulty, we assumed full susceptibility of the population and made a crude adjustment on the estimates of transmission rates, which will be described in the subsection of *Post-cessation simulation*.

In post-cessation simulations, children under 5 were born post-cessation and vaccinated only with IPV, which is known to provide limited protection against infection [2, 4]. In alignment with estimate from the literature [2], we assumed 80% of children under 5 were susceptible to poliovirus. As to individuals born pre-cessation, we expected their intestinal immunity induced by WPV and/or OPV to have gradually waned, and initialized each person to susceptible state with a probability of 0.8, and to recovered state with the remaining probability. We varied this probability in sensitivity analysis.

*Transmission model*

We use a parameter $\beta_{i,j}^{(w)}$ to denote the probability per day of a susceptible individual acquiring serotype *i* (*i* =1, 2, 3) from an infected individual within the same household in community *j* (*j*=C, H, T representing Capoluca, Campo Grande, and Tuxpanguillo, respectively), and $\beta_{i,j}^{(b)}$ denote that probability from someone outside the household. The hazard of serotype *i* infection for a susceptible individual in community *j* at time *t* is then given by $\beta_{i,j}^{\left( w \right)}I_{i,j}^{\left( w \right)}\left( t \right)+\beta_{i,j}^{\left( b \right)}I_{i,j}^{\left( b \right)}(t)$, where $I_{i,j}^{\left( w \right)}\left( t \right)$ and $I_{i,j}^{\left( b \right)}(t)$ represent the number of people shedding within and outside this individual’s household, respectively. We further multiply this hazard by $\alpha$ (an unknown constant greater than 1) if the susceptible is under 5 years old, because younger children are considered less hygienic relative to older children and adults, thus more likely to acquire infection fecal-orally [5].

*Model fitting*

To avoid overfitting, we introduced the following assumptions to limit the number of unknown model parameters. First, average latency duration does not differ between vaccinees and non-vaccinees, nor between IPV-only cohort and OPV exposed cohort. Second, all community members shared the same immunity profile and were vaccinated with OPV before. Third, we assume $\frac{\beta_{1,j}^{\left( w \right)}}{\beta_{2,j}^{\left( w \right)}}=\frac{\beta_{1,j}^{\left( b \right)}}{\beta_{2,j}^{\left( b \right)}}=$ $\gamma_{1}$, and $\frac{\beta_{3,j}^{\left( w \right)}}{\beta_{2,j}^{\left( w \right)}}=\frac{\beta_{3,j}^{\left( b \right)}}{\beta_{2,j}^{\left( b \right)}}=\gamma_{3}$ for any *j* (*j=*C, H, T); in other words, relative differences in transmission between strains are consistent across communities. Note that the transmission rates capture effective contact rates as well as the intrinsic transmissibility of a virus. This assumption essentially disentangles these two components, and the two ratios $\gamma_{1}$ and $\gamma_{3}$ reflect the relative infectivity of OPV1 and OPV3 compared to OPV2. Table S1 summarizes the set of model parameters to be estimated and their respective biological interpretations.

Households not enrolled in the study were also included in our model, even though they provided no stool samples thus remained unobserved throughout the study. To reduce the cost of computation in model fitting, we tracked the aggregate number of infectious people in unobserved households instead of the state of each individual. Utilizing the stool shedding data in observed unvaccinated households, we fitted a logistic regression model to predict whether an unvaccinated household would have at least one shedding event during the study period based on the number of children under 5 in that household. The odds ratio was 1.84, 1.56, and 1.44 for type 1, 2, and 3 OPV, respectively. At each time step and for each serotype, we could obtain a crude estimate of the total number of infectious people in unobserved (thus unvaccinated) households by adjusting its counterpart in observed unvaccinated households with the odds ratio and the difference in average numbers of young children in these two classes of households.

We followed the ABC Sequential Monte Carlo (SMC) approach developed by Toni *et al.* [6] to fit our model. The priors of model parameters were set as independent uniform distributions, with ranges chosen to be weakly informative (Table S1). To be specific, we made 1) maximum likelihood estimations (MLE) of transmission rates ($\beta_{2,j}^{(w)}$ and $\beta_{2,j}^{(b)}$, for *j*=C, H, T) using the first time point of stool shedding data of unvaccinated individuals, 2) MLE of latency duration using the first time point of stool shedding data of vaccine recipients, and 3) crude estimates of average shedding durations ($1/\sigma_{i}^{v}$and $1/\sigma_{i}^{n}$, for *i*=1, 2, 3) by assuming the beginning and end of shedding as the mid-point between stool samples. We then used these estimates to come up with reasonable ranges to regularize the subsequent inferences of the corresponding parameters. As to the remaining three unknown parameters ($\alpha$, $\gamma_{1}$, $\gamma_{3}$), ranges were elicited from assessment of experienced experts. A population of 5,000 particles were sampled from the prior distribution, propagated through a sequence of intermediate distributions that gradually evolve towards the target posterior distribution. To efficiently compress the information contained in observed dataset, the summary statistics were designed to be the fraction of individuals shedding in three groups – vaccinees, household contacts of vaccinees, and unvaccinated household members – on days when at least one third of the individuals in the group provided stool samples. We used *L*1 distance to measure the similarity between observed and simulated summary statistics, and chose tolerance to be the lower quartile of the distances corresponding to the previous generation of particles. Propagation was ended after 15 rounds of evolution.

*Post-cessation simulations*

Each simulation is run using a set of model parameters randomly drawn from the posterior distribution obtained in model fitting. Note that our assumption of a fully susceptible population when fitting the model inevitably leads to an underestimation in transmission rates. In the Mexico study, approximately 60% of the vaccinees responded to OPV challenge, so we employed this fraction as a rough estimate of the population level susceptibility and adjusted $\beta_{2,j}^{(w)}$ and $\beta_{2,j}^{(b)}$ (*j*=C, H, T) by 1/0.6, which were then used for post-cessation simulations. Also, we had no inference regarding the shedding duration in IPV-only cohort, here we adopted estimations from publications [2] and assumed vaccinees to shed 20 days on average after OPV challenge, while naturally infected individuals shed for half as long.

**Supplemental Results**

Table S1: Description and estimate of model parameters.

| **Parameter** | **Description** | **Range of Prior** | **Estimate (95% CI)** |
| --- | --- | --- | --- |
| $\beta_{2,C}^{(w)}$  $\beta_{2,H}^{(w)}$  $\beta_{2,T}^{(w)}$ | Within household transmission rate (${\times10}^{-3}$ per contact day)  of serotype 2 in Capoluca, Campo Grande, and Tuxpanguillo | (0.1, 10) | 5.56 (4.68, 5.96)  7.54 (6.52, 7.99)  1.53 (0.71, 3.04) |
| $\beta_{2,C}^{\left( b \right)}$  $\beta_{2,H}^{\left( b \right)}$  $\beta_{2,T}^{\left( b \right)}$ | Between household transmission rate (${\times10}^{-5}$ per contact day) of serotype 2 in Capoluca, Campo Grande, and Tuxpanguillo | (0.1, 10) | 2.50 (1.57, 2.91)  2.14 (1.41, 2.52)  2.04 (1.44, 2.75) |
| $\alpha$ | Relative susceptibility of children under 5  compared to people above 5 | (1, 10) | 2.85 (2.08, 3.65) |
| $\gamma_{1}$  $\gamma_{3}$ | Relative transmissibility of serotype 1, 3  compared to serotype 2 | (0, 2) | 0.36 (0.21, 0.63)  0.75 (0.59, 0.90) |
| $1/\lambda_{1}$  $1/\lambda_{2}$  $1/\lambda_{3}$ | Average latency duration (in days) of serotype 1, 2, and 3 | (1, 5) | 1.9 (1.3, 3.1)  2.5 (1.5, 3.6)  2.3 (1.1, 4.1) |
| $1/\sigma_{1}^{v}$  $1/\sigma_{2}^{v}$  $1/\sigma_{3}^{v}$ | Average shedding duration (in days) of serotype 1, 2, and 3  in vaccinees exposed to OPV before | (5, 20) | 9.8 (9.2, 11.0)  10.2 (9.5, 11.4)  14.5 (12.7, 17.0) |
| $1/\sigma_{1}^{n}$  $1/\sigma_{2}^{n}$  $1/\sigma_{3}^{n}$ | Average shedding duration (in days) of serotype 1, 2, and 3  in non-vaccinees exposed to OPV before | (3, 15) | 7.3 (6.7, 8.6)  7.8 (6.7, 9.1)  9.9 (8.4, 11.7) |

Figure S1: Simulated outbreak response using OPV in Campo Grande 5 years after cessation.

Figure S2: Simulated outbreak response using OPV in Tuxpanguillo 5 years after cessation.

Figure S3: Simulated silent circulation triggered by one infectious case in Campo Grande 5 years after cessation.


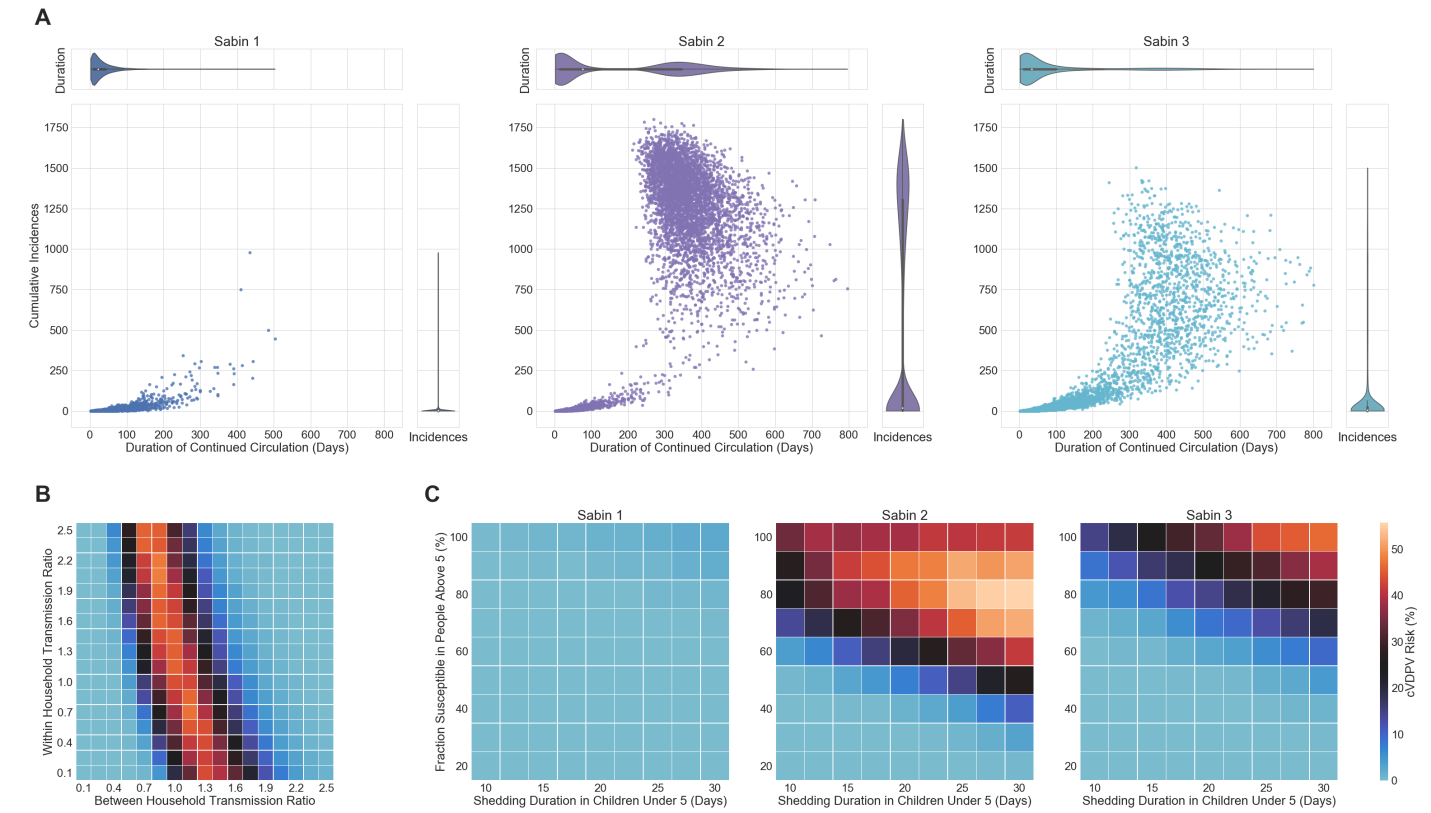


Figure S4: Simulated silent circulation triggered by one infectious case in Tuxpanguillo 5 years after cessation.


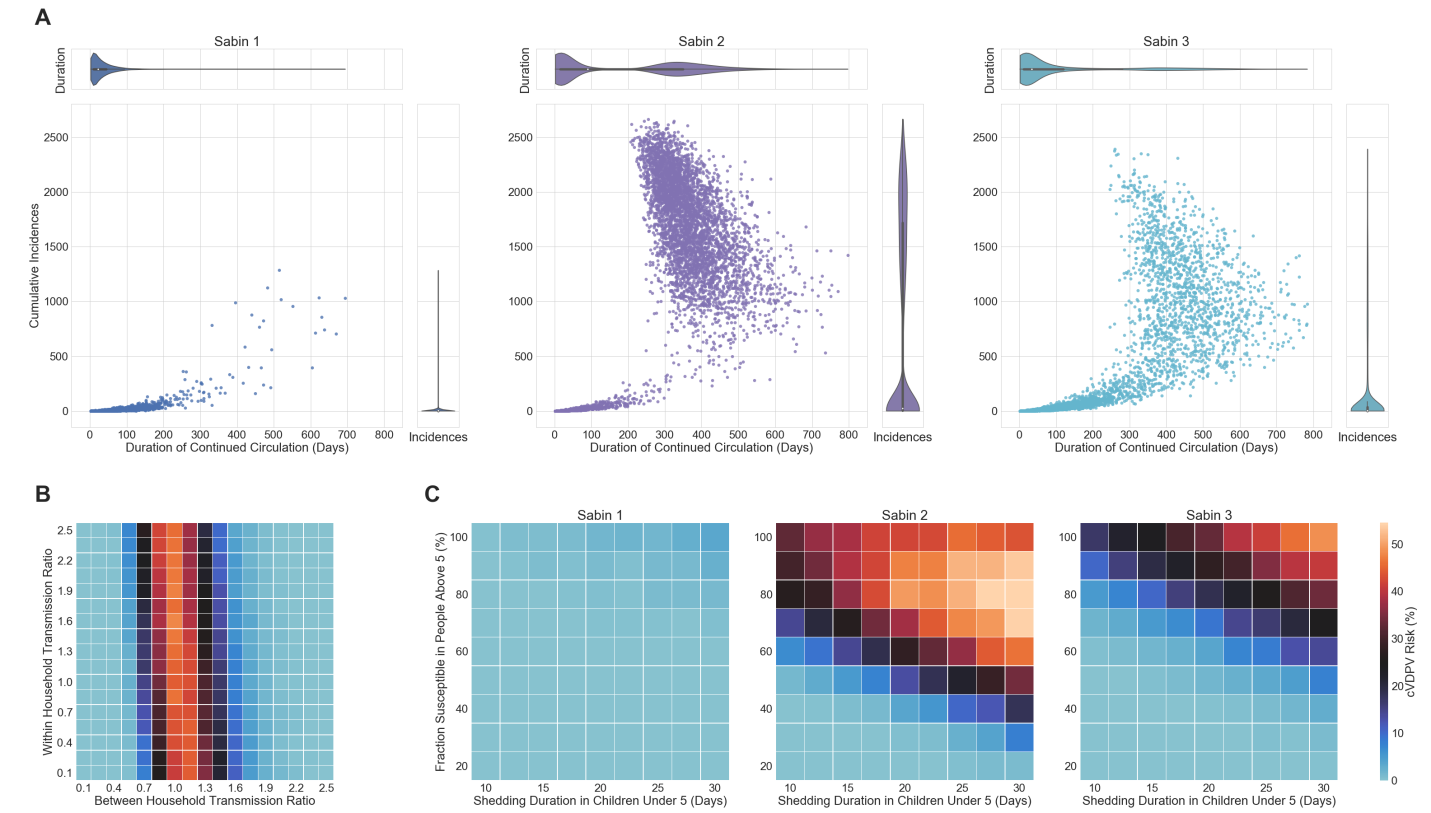


**References**

1. Sarnquist C. Poliovirus vaccine transmissibility in communities after cessation of routine oral poliovirus vaccine immunization. **2018**.

2. Duintjer Tebbens RJ, Pallansch MA, Chumakov KM, et al. Review and assessment of poliovirus immunity and transmission: synthesis of knowledge gaps and identification of research needs. Risk analysis : an official publication of the Society for Risk Analysis **2013**; 33(4): 606-46.

3. Dowdle WR, Birmingham ME. The biologic principles of poliovirus eradication. J Infect Dis **1997**; 175 Suppl 1: S286-92.

4. Duintjer Tebbens RJ, Pallansch MA, Chumakov KM, et al. Expert review on poliovirus immunity and transmission. Risk analysis : an official publication of the Society for Risk Analysis **2013**; 33(4): 544-605.

5. Gelfand HM, Potash L, LeBlanc DR, Fox JP. Intrafamilial and interfamilial spread of living vaccine strains of polioviruses. Journal of the American Medical Association **1959**; 170(17): 2039-48.

6. Toni T, Welch D, Strelkowa N, Ipsen A, Stumpf MPH. Approximate Bayesian computation scheme for parameter inference and model selection in dynamical systems. Journal of The Royal Society Interface **2009**; 6(31): 187.
